# NRF2 Plays a Crucial Role in the Tolerogenic Effect of Ethyl Pyruvate on Dendritic Cells

**DOI:** 10.3390/ijms25116195

**Published:** 2024-06-04

**Authors:** Suzana Stanisavljević, Goran Stegnjaić, Bojan Jevtić, Mirjana Dimitrijević, Đorđe Miljković, Irena Lavrnja, Neda Nikolovski

**Affiliations:** 1 Department of Immunology, Institute for Biological Research “Siniša Stanković”, National Institute of Republic of Serbia, University of Belgrade, 11060 Belgrade, Serbia; ssuzana@ibiss.bg.ac.rs (S.S.); goran.stegnjaic@ibiss.bg.ac.rs (G.S.); bojan.jevtic@ibiss.bg.ac.rs (B.J.); mirjana.dimitrijevic@ibiss.bg.ac.rs (M.D.); neda.djedovic@ibiss.bg.ac.rs (N.N.); 2 Department of Neurobiology, Institute for Biological Research “Siniša Stanković”, National Institute of Republic of Serbia, University of Belgrade, 11060 Belgrade, Serbia; irenam@ibiss.bg.ac.rs

**Keywords:** ethyl pyruvate, dendritic cells, immunotherapy, autoimmunity, NRF2, NF-kappaB

## Abstract

Ethyl pyruvate (EP) is a redox-active compound that has been previously shown to be effective in restraining immune hyperactivity in animal models of various autoimmune and chronic inflammatory diseases. Importantly, EP has also been proven to have a potent tolerogenic effect on dendritic cells (DCs). Here, the influence of EP on the signaling pathways in DCs relevant for their tolerogenicity, including anti-inflammatory NRF2 and pro-inflammatory NF-κB, was explored. Specifically, the effects of EP on DCs obtained by GM-CSF-directed differentiation of murine bone marrow precursor cells and matured under the influence of lipopolysaccharide (LPS) were examined via immunocytochemistry and RT-PCR. EP counteracted LPS-imposed morphological changes and down-regulated the LPS-induced expression of pro-inflammatory mediators in DCs. While it reduced the activation of NF-κB, EP potentiated NRF2 and downstream antioxidative molecules, thus implying the regulation of NRF2 signaling pathways as the major reason for the tolerizing effects of EP on DCs.

## 1. Introduction

Ethyl pyruvate (EP) is a redox-active compound that is readily taken up by cells and exerts a profound influence on metabolism, intracellular signaling and mRNA expression. EP is a redox analog of dimethyl fumarate (Tecfidera), an approved therapeutic agent for multiple sclerosis [1]. EP shows a strong immunomodulatory effect in various animal models of autoimmune and inflammatory disorders [2]. The dominant immunomodulatory effect of EP has been observed in T cells, macrophages, and dendritic cells (DCs). For instance, EP ameliorated type 1 diabetes (T1D) in mice by potentiating the function of regulatory T cells (Treg) and tolerogenic DCs [3]. EP has also been shown to ameliorate experimental autoimmune encephalomyelitis (EAE), an animal model of CNS autoimmunity, with suppression achieved by limiting encephalitogenic T cells and macrophages [4,5]. Direct evidence for EP’s tolerogenic effects on DCs was obtained in our study on murine and human DCs in vitro [6]. The addition of EP to cultures of mouse bone-marrow-derived monocytes and human peripheral blood monocytes during the differentiation to DCs led to a marked decrease in the expression of MHC class II molecules and co-stimulatory molecules CD86 and CD83 in DCs exposed to maturation stimuli [6]. Consequently, these cells had a limited ability to stimulate an allogeneic T cell response. Importantly, the effect of EP on human cell cultures was comparable to that of the known tolerogenic agent vitamin D3 [6]. It is also worth noting that the effects on human cells were measured not only with the cells from healthy individuals but also from patients with multiple sclerosis, which supports EP’s potential therapeutic applicability. In another study, EP was shown to promote the tolerogenic properties of DCs by down-regulating the expression of maturation-related Toll-like receptors (TLRs) 7 and 9 through a reduction in glycolysis and mitochondrial respiration in DCs [7].

Our previous studies have shown that EP is a powerful immunomodulatory compound with the potential to be used as a tolerizing agent for DC-based cell therapy [2,6]. The aim of this study was to further characterize the tolerogenic effect of EP on DCs in order to take a step towards the goal of using EP-treated DCs as cell therapeutics. To this end, an examination of EP’s influence on cell morphology and mRNA expression of pro- and anti-inflammatory molecules was performed in mouse DCs. Furthermore, the intracellular mechanisms behind the tolerogenic effects of EP on DCs were investigated. We were particularly interested in the pro-inflammatory transcription factor nuclear factor-κB (NF-κB) and antioxidant signaling pathways controlled by nuclear factor erythroid 2-related factor 2 (NRF2).

## 2. Results

**Figure 1:**
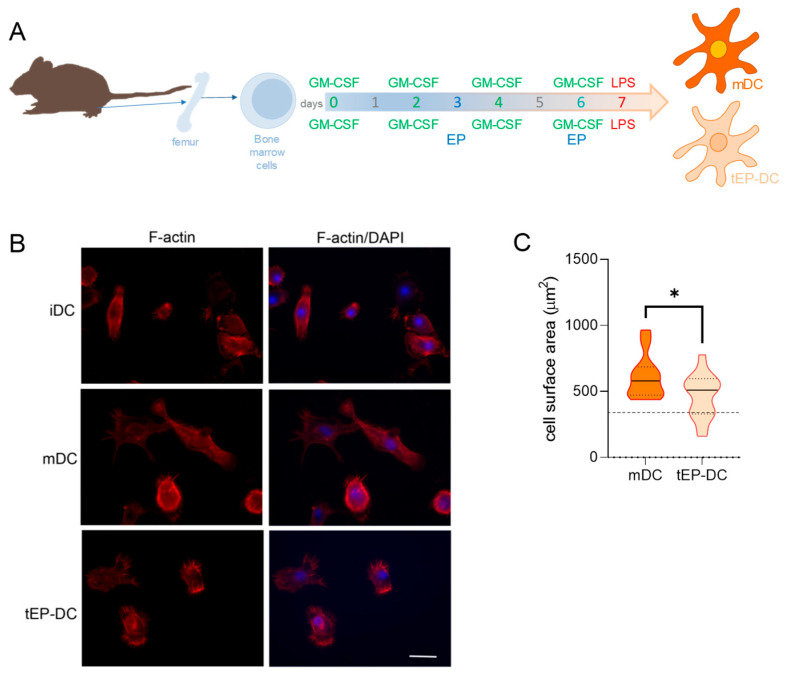
Effects of EP on morphological characteristics of DCs. BMDCs were obtained from C57BL/6 mice and differentiated towards DCs in the presence of GM-CSF (20 ng/mL). Cells were stimulated with LPS (100 ng/mL) for 4 h without previous EP treatment (mDCs) or previously treated with 3.1 mM EP (tEP-DCs) (**A**). Immunocytochemistry was used to stain actin filaments with phalloidin (red) and with DAPI nuclear stain to visualize the nuclei (blue). Representative micrographs are shown. The scale bar is 20 µm (white line) (**B**). The results obtained in two experiments are presented as a violin plot (n = 12); the dashed line represents the level obtained in iDCs (**C**). * *p* < 0.05, *t*-test.

To determine the effects of EP on the morphology and size of DCs, staining of actin filaments was performed. An irregular shape with a long dendritic extension, with capacity for antigen presentation and T cell priming, is a characteristic feature of immunogenic DCs. On the other hand, a round and smaller cell body without dendrites is a feature of immature DCs (iDC) that are unable to induce an immune response [8]. While mDCs exhibited a star-like form with protruding dendrites, DCs treated with EP had a small cell body without dendrites (Figure 1). This was quantified in our study as the cell surface area, where a larger area indicates a more irregular shape, while a smaller area implies a more rounded shape. Accordingly, tEP-DCs were significantly smaller compared to mDCs and morphologically resembled immature DCs, thus supporting our previous finding that EP tolerizes DCs. 

**Figure 2:**
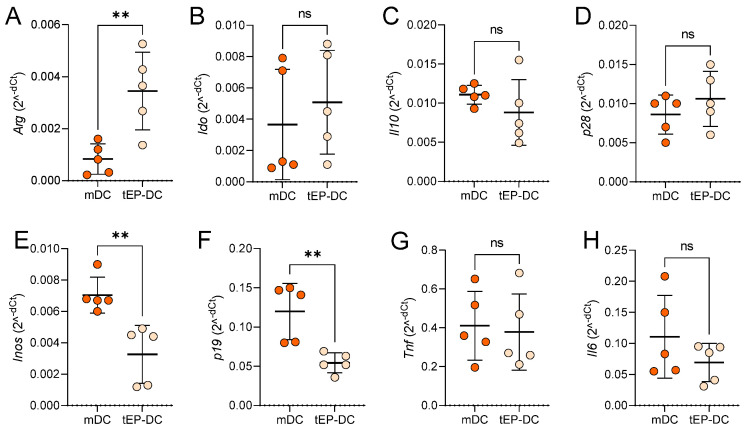
Relative mRNA levels of inflammation-related genes in DCs. BMDCs were obtained from C57BL/6 mice and differentiated towards DCs in the presence of GM-CSF (20 ng/mL). Cells were stimulated with LPS (100 ng/mL) for 2 h without previous EP treatment (mDCs) or previously treated with 3.1 mM EP (tEP-DCs). Relative mRNA expression of *Arg* (**A**), *Ido* (**B**), *Il10* (**C**), *p28* (**D**), *Inos* (**E**), *p19* (**F**), *Tnf* (**G**), and *Il6* (**H**) was determined in DCs by RT-PCR. Data are presented as individual values and as mean +/− SD, n = 5. ** *p* < 0.01, *t*-test.

To further characterize the tolerogenic influence of EP on DCs, mRNA levels of anti-inflammatory and pro-inflammatory mediators were compared in mDCs and tEP-DCs. The expression of mRNA for anti-inflammatory arginase was significantly up-regulated in tEP-DCs (Figure 2A), while the expression of mRNA for pro-inflammatory inducible NO synthase (iNOS) and interleukin (IL)-23 subunit p19 was reduced in tEP-DCs (Figure 2E,F). mRNA expression of indoleamine 2,3-dioxygenase (IDO), IL-10, IL-27 subunit p28, TNF, and IL-6, did not differ significantly between mDCs and tEP-DCs (Figure 2B–D,G,H).

**Figure 3:**
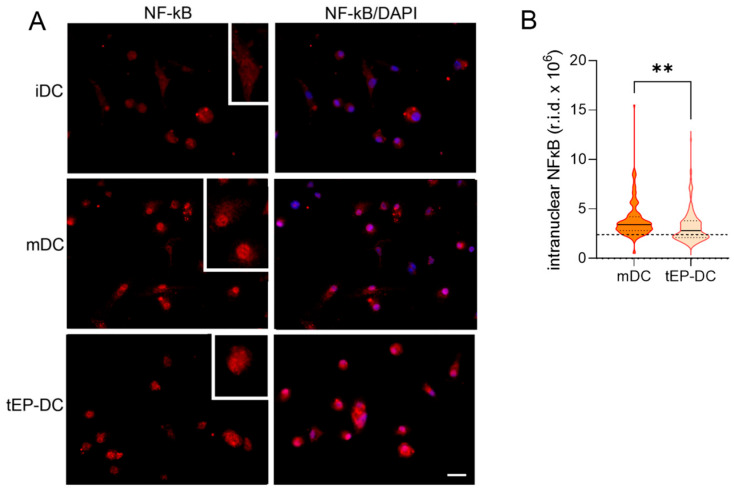
Effect of EP on NF-κB activation in DCs. BMDCs were obtained from C57BL/6 mice and differentiated towards DCs in the presence of GM-CSF (20 ng/mL). Cells were stimulated with LPS (100 ng/mL) for 1 h without previous EP treatment (mDCs) or previously treated with 3.1 mM EP (tEP-DCs). Immunocytochemistry was used to stain cells with an antibody against NF-κB (red) and with DAPI nuclear stain to visualize the nucleus (blue). Representative micrographs with insets (twice enlarged) are shown (**A**). The scale bar is 20 µm (white line). The results obtained in two experiments are presented as a violin plot (n = 150); the dashed line represents the level obtained in iDCs (**B**). r.i.d.—raw integrated density; ** *p* < 0.01, *t*-test.

**Figure 4:**
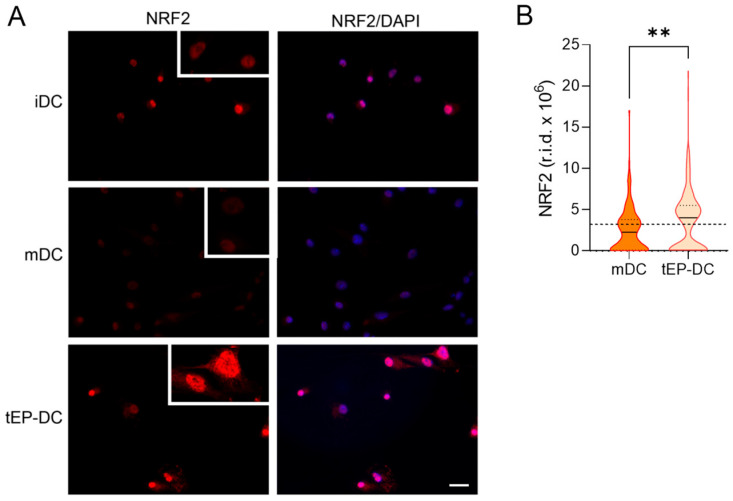
Effect of EP on NRF2 activation in DCs. BMDCs were obtained from C57BL/6 mice and differentiated towards DCs in the presence of GM-CSF (20 ng/mL). Cells were stimulated with LPS (100 ng/mL) for 2 h without previous EP treatment (mDCs) or previously treated with 3.1 mM EP (tEP-DCs). Immunocytochemistry was used to stain cells with an antibody against NRF2 (red) and with DAPI nuclear stain to visualize the nucleus (blue). Representative micrographs with insets (twice enlarged) are shown (**A**). The scale bar is 50 µm (white line). The results obtained in two experiments are presented as a violin plot (n = 150); the dashed line represents the level obtained in iDCs (**B**). r.i.d.—raw integrated density; ** *p* < 0.01, *t*-test.

To explore the intracellular signaling behind the tolerogenic effects of EP, dendritic cells were stained with specific antibodies against pro-inflammatory NF-κB or anti-inflammatory NRF2, together with DAPI nuclear stain. The intranuclear staining intensity of NF-κB was reduced in tEP-DCs in comparison to mDCs (Figure 3). The opposite was observed for NRF2 (Figure 4).

**Figure 5:**
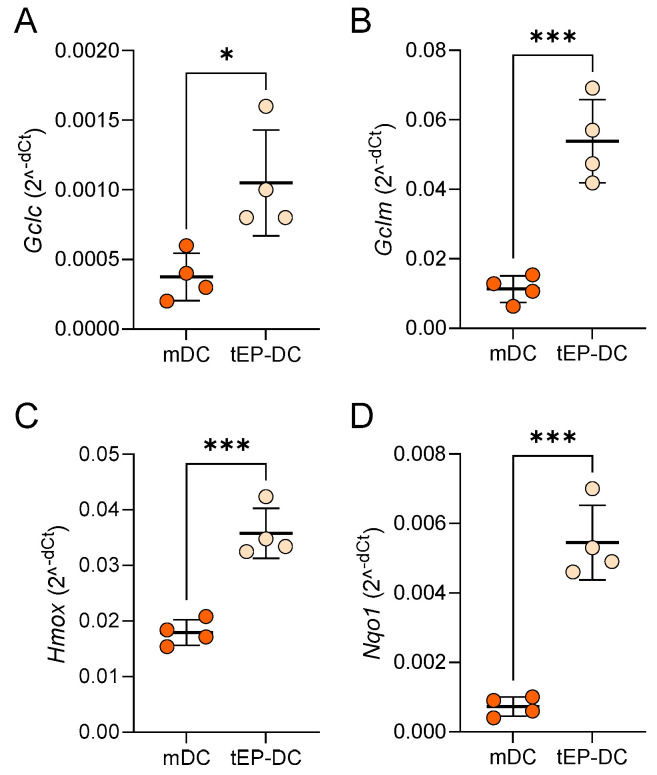
Relative mRNA level of Nrf2-regulated genes in DCs. BMDCs were obtained from C57BL/6 mice and differentiated towards DCs in the presence of GM-CSF (20 ng/mL). Cells were stimulated with LPS (100 ng/mL) for 2 h without previous EP treatment (mDCs) or previously treated with 3.1 mM EP (tEP-DCs). Relative mRNA expression of *Gclc* (**A**), *Gclm* (**B**), *Hmox* (**C**), and *Nqo1* (**D**) was determined in DCs by RT-PCR. Data are presented as mean +/− SD, n = 4. * *p* < 0.05, *** *p* < 0.001, *t*-test.

To further explore the effect of EP on NRF2 signaling, the mRNA levels of genes downstream from NRF2 were determined in DCs. Notably, the mRNA expression of glutamate–cysteine ligase catalytic subunit (GCLC), glutamate–cysteine ligase regulatory subunit (GCLM), heme oxygenase (HO-1) and NAD(P)H dehydrogenase quinone 1 (NQO1) was markedly increased in tEP-DCs (Figure 5).

**Figure 6:**
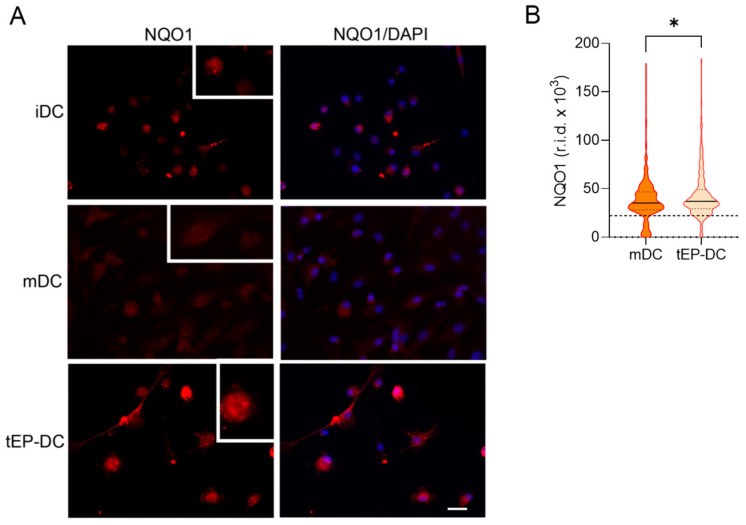
Effect of EP on NQO1 activation in DCs. BMDCs were obtained from C57BL/6 mice and differentiated towards DCs in the presence of GM-CSF (20 ng/mL). Cells were stimulated with LPS (100 ng/mL) for 2 h without previous EP treatment (mDCs) or previously treated with 3.1 mM EP (tEP-DCs). (**A**) Immunocytochemistry was used to stain cells with an antibody against NQO1 (red) and with DAPI nuclear stain to visualize the nucleus (blue). Representative micrographs with insets (twice enlarged) are shown (**A**). The scale bar is 20 µm (white line). The results obtained in three experiments are presented as a violin plot (n = 300); the dashed line represents the level obtained in iDCs (**B**). r.i.d.—raw integrated density; * *p* < 0.05, *t*-test.

**Figure 7:**
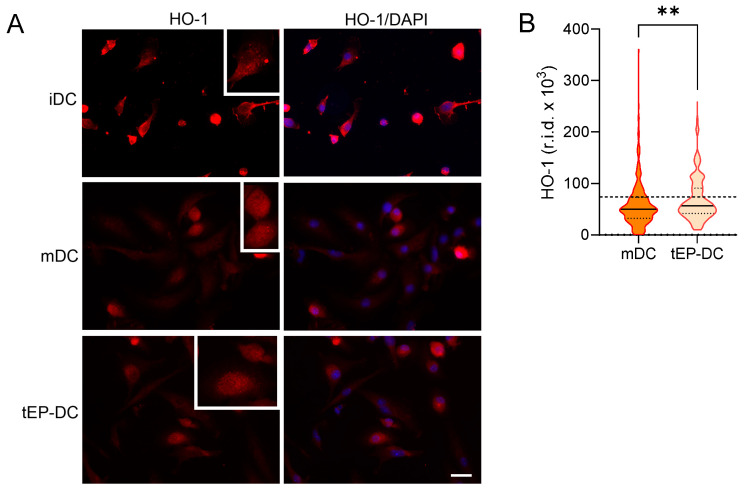
Effect of EP on HO-1 activation in DCs. BMDCs were obtained from C57BL/6 mice and differentiated towards DCs in the presence of GM-CSF (20 ng/mL). Cells were stimulated with LPS (100 ng/mL) for 2 h without previous EP treatment (mDCs) or previously treated with 3.1 mM EP (tEP-DCs). (**A**) Immunocytochemistry was used to stain cells with an antibody against HO-1 (red) and with DAPI nuclear stain to visualize the nucleus (blue). Representative micrographs with insets (twice enlarged) are shown (**A**). The scale bar is 20 µm (white line). The results obtained in three experiments are presented as a violin plot (*n* = 300); the dashed line represents the level obtained in iDCs (**B**). r.i.d.—raw integrated density; ** *p* < 0.01, *t*-test.

Finally, DC cultures were stained with specific antibodies against NQO1 and HO-1 together with DAPI. The staining intensity of both NQO1 and HO-1 was increased in tEP-DCs compared to mDCs (Figure 6 and Figure 7).

## 3. Discussion

This study shows that EP potentiates gene expression of anti-inflammatory arginase I and reduces gene expression of pro-inflammatory iNOS and IL-23 in DCs. Up-regulation of antioxidative NRF2 signaling in DCs is observed in parallel. These results extend our previous observations of the tolerogenic influence of EP on DCs. Namely, our previous data have shown that EP potently down-regulates the release of various pro-inflammatory cytokines from DCs and reduces the expression of molecules involved in antigen presentation [6]. Also, the results of this study are in accordance with the ability of EP to reduce the production of pro-inflammatory cytokines, NO and reactive oxygen species, as well as the expression of MHC II molecules in myeloid populations, i.e., in peritoneal and bone-marrow-derived macrophages, as shown in our earlier paper [4]. 

Arginase I activity in DCs has been linked to their tolerogenic properties [9,10]. Likewise, the decreased iNOS expression in DCs is in accordance with the results previously obtained in EP-treated macrophages [3,11], as well as in DCs treated with classical DC-tolerizing agents of glucocorticoids and vitamin D3 [12,13,14]. Activated DCs are one of the main cellular sources of IL-23 [15]. DCs transfected with antisense oligonucleotides against IL-23p19 had a markedly reduced ability to stimulate proliferation of allogeneic CD4^+^ T lymphocytes [16]. Thus, down-regulation of IL-23p19 mRNA under the influence of EP might contribute to its tolerizing effect on DCs. On the contrary, vitamin D3 potentiated IL-23, TNF, and IL-6 in DCs [17,18]. While TNF and IL-6 were unaffected in our system, glucocorticoid-treated cells exhibited a lower TNF production [19,20,21,22]. The observed variance in the effects of the tolerizing agents on cytokine production in DCs could be a consequence of different protocols for DC generation and maturation, as well as of the different timing of the agent’s application. Further, the lack of effect of EP on anti-inflammatory IL-10, IL-27, and IDO, as well as on pro-inflammatory TNF and IL-6, might be specific for the early time point that we investigated in our study. Accordingly, IL-6 and TNF reductions in tEP-DCs were observed in our previous study when the levels of these cytokines were measured 24 h after LPS stimulation [6].

One of the key pro-inflammatory transcription factors involved in cell differentiation, apoptosis, and the body’s defense is NF-κB [23]. Importantly, activation of NF-κB induces the maturation of DCs [23], increases their ability to produce pro-inflammatory cytokines [24], and has been identified as a major factor in DC immunogenicity [25]. In line with this, the tolerogenic effect of vitamin D3 or glucocorticoids on DCs was associated with their ability to reduce the activation of NF-κB [12,26,27,28,29,30]. Accordingly, the intensity of NF-κB staining in cell nuclei, representing active NF-κB, was reduced in tEP-DCs compared to mDCs, suggesting that the anti-inflammatory effects of EP on DCs are caused, at least in part, by inhibition of NF-κB signaling. In addition, various in vitro and in vivo studies demonstrated the ability of EP to inhibit NF-κB DNA binding [31,32,33,34,35]. Thus, inhibition of NF-κB DNA binding might also be important for the tolerogenic effect of EP on DCs and should be explored in future studies.

The functions of DCs, such as cell maturation, activation, and cytokine production, are also highly dependent on intracellular redox homeostasis and the generation of reactive oxygen species (ROS) [36]. NRF2 is a key transcription factor that regulates the transcription of antioxidant and cell-protective genes [37], and as such has a significant role in the maintenance of redox homeostasis [9] and counteracting the increase in intracellular ROS [36]. During the increase in ROS generation in DCs, NRF2 translocates into the nucleus and binds to the antioxidant response elements, leading to activation of its target genes such as *Hmox* and *Nqo1*, which are associated with ROS detoxification [10]. HO-1 is a rate-limiting enzyme [38] that inhibits heme synthesis, leading to increased carbon monoxide (CO) production and inhibition of DC activity [39,40]. Accordingly, HO-1 has been shown to be of great importance for the induction of Treg by DCs [40,41]. Also, an increased expression of NQO1 is responsible for cell protection from ROS [42,43]. The effect of vitamin D3 on NRF2, HO-1 and NQO1 has not been examined in DCs so far, while glucocorticoids are well known for their inhibitory effect on these molecules [44,45,46]. Importantly, we showed previously that EP exerts comparable immunomodulatory effects to dimethyl fumarate (DMF) [4], a well-known Nrf2 activator, and the principle pharmaceutical active ingredient of Tecfidera and Skilarence, i.e., drugs for the autoimmune diseases of multiple sclerosis and psoriasis, respectively. EP can be considered as a redox analogue of DMF, and its activity is partly achieved by redox reactivity [2]. Thus, the redox activity of EP and its potent promotion of NRF2 signaling could be responsible for the potential specificities of tEP-DCs in comparison to tolDCs obtained under the influence of vitamin D3 or glucocorticoids.

Activation of the NRF2 signaling pathway causes a decrease in pro-inflammatory cytokine production in DCs, indicating that NRF2 is one of the key factors in the induction and differentiation of tolDCs [37]. Indeed, a deficiency of NRF2 in DCs leads to their increased maturation and immunogenicity [36,47], and decreased NRF2 levels and impaired ROS clearance were observed in DCs of systemic lupus erythematosus patients [48]. Accordingly, NRF2-mediated metabolic reprogramming of DCs led to tolerogenic activity in a murine model of aplastic anemia [37]. Interestingly, dimethyl fumarate, a redox analog of EP, was shown to activate the NRF2 transcription pathway in DCs of multiple sclerosis patients and to increase the frequency of Treg in their peripheral blood [49].

The following are some of the limitations of the present study. This study examined early time points after the maturation stimulus LPS was added to DC cultures. The idea behind such an approach was to determine the effects of EP on early maturation processes in DCs, as later time points for cytokine production were examined in our previous study [6]. Still, this is the first study on EP’s effects on intracellular signaling and only one time point was examined for each of the readouts. This is clearly a limitation of the present study, as different outcomes might be observed at alternative time points. Further, the antioxidant activity was not determined in DCs in this study. Our previous results show that EP is able to down-regulate reactive oxygen species and NO production and act as a scavenger of superoxide and hydroxyl radicals [4]. Thus, it is important to determine the antioxidant effects of EP in DCs in the future. Also, the expression of inflammatory mediators was only determined at the transcriptional level, and not at the level of protein products. However, the LPS-induced inflammatory response is highly dependent on transcriptional regulation [50], thus implying that similar effects would be observed at the protein level. Finally, our results present only an association between Nrf2 activation and anti-inflammatory properties in DCs, as Nrf2 inhibitors were not used in this study. Hence, the final confirmation of the essential role of Nrf2 in DC tolerogenicity has to be obtained in forthcoming studies with Nrf2 inhibitors.

## 4. Materials and Methods

### 4.1. Bone-Marrow-Derived Dendritic Cells

Murine DCs were obtained from progenitor bone marrow cells that were flushed from the femur of C57BL/6 female mice (experiments were approved by the local ethics committee of the Institute for Biological Research “Siniša Stanković” in accordance with Directive 2010/63/EU, N° 03-01/17 and No 02-4/19). These cells were cultured in RPMI 1640 (Capricorn Scientific, Ebsdorfergrund, Germany) supplemented with 20% FCS (PAA Laboratories), 2 mM glutamine and 1 mM sodium pyruvate (both from Sigma-Aldrich (St. Louis, MO, USA)) (1 × 10^6^/mL/well in a 24-well plate). Bone-marrow-derived dendritic cells were cultivated for 7 days in the presence of 20 ng/mL of GM-CSF (Peprotech or Novus, Littleton, CO, USA). Treatment with 3.1 mM of EP was performed on days 3 and 6 of cultivation to produce tolerogenic DCs (tEP-DCs). An EP concentration of 3.1 mM and the timing of its application during the differentiation of DCs from bone marrow were chosen in this study in accordance with the results of our previous study investigating the dose- and time-dependence of the effects of EP on DCs [6]. Alternatively, the cells were not treated with EP, but with vehicle (mDCs). A total of 100 ng/mL lipopolysaccharide (LPS, Sigma-Aldrich), as a maturation stimulus, was added at the end of cultivation. DCs that were not treated with EP and not stimulated with LPS were immature DCs (iDCs).

DCs were characterized via flow cytometry on a CytoFLEX flow cytometer (Beckman Coulter, Indianapolis, IN, USA), and analyzed using CytExpert 2.4.0.28 software (Beckman Coulter). The following antibodies (all from Thermo Fisher Scientific, Waltham, MA, USA) were used: AF488-conjugated anti-mouse CD11b (M1/70), APC-conjugated anti-mouse MHC II (M5/114.15.2), PE-Cy5-conjugated anti-mouse CD86 (GL1), eFluor 450-conjugated anti-mouse/rat CD40 (HM40-3), and eFluor 506-conjugated anti-mouse CD11c (N418). The percentages of positivity after 24 h of DC cultivation with LPS, presented as means +/- SD, were CD11b (95.3+/−0.2), CD11c (54.0+/−6.0), MHC class II (72.3+/−4.0), CD40 (63.6+/−5.8), and CD86 (47.6+/−4.2). Adequate isotype control antibodies were used where necessary to set the gates for cell marker positivity. Typically, the proportion of isotype control antibody-stained cells was <1%. The results of cytofluorimetry are presented as the percentage of cells.

DCs were exposed to LPS for 4 h (for NQO1 and HO-1 staining), 2 h (for NRF2 and actin filament staining, as well as for RT-PCR analysis) or 1 h (for NF-κB staining). The number of viable cells was determined via the trypan blue exclusion test on a LUNA-II™ Automated Cell Counter from Logos Biosystems (Anyang-si, Republic of Korea).

### 4.2. Phalloidin Staining

For actin filament visualization, phalloidin staining was used to determine the size and morphology of DCs. After treatment, cells were fixed with 4% paraformaldehyde for 20 min at 4 °C and subsequently washed three times with PBS and permeabilized with 0.25% Triton X-100 (Sigma-Aldrich) for 10 min. After the time had expired, cells were washed with PBS again and unspecific staining was blocked with 3% bovine serum albumin (Sigma-Aldrich) for 30 min at room temperature. F-actin was stained with ActinRed 555 ReadyProbes (dilution 1:50 in PBS; Thermo Fisher Scientific) for 30 min at room temperature in the dark. After washing with PBS and distilled water, samples were stained with 4′,6-Diamidino-2-Phenylindole (DAPI) (Invitrogen) for 5 min. Coverslips were mounted with Mowiol (Calbiochem, Darmstadt, Germany) and cells were observed using a Zeiss Axiovert fluorescent microscope (Zeiss, Jena, Germany). Images were analyzed using AxioVisionRel 4.9.1 software (Zeiss) to quantify the cell surface area in each group. The mean cell surface area was determined in 6 areas per chamber slide and the results are expressed in µm^2^.

### 4.3. Immunofluorescent Labeling for NF-κB, NRF2, NQO1, and HO-1

After fixing, washing and permeabilizing the cells as previously described, unspecific staining was blocked with 3% bovine serum albumin (Sigma-Aldrich) for 30 min at room temperature. Primary antibodies against NF-κB/p-65, NRF2, NQO1 and HO-1 (all purchased from Santa Cruz, Dallas, TX, USA) were applied overnight at 4 °C. After washing with PBS on the next day, the cells were incubated with appropriate secondary antibodies for 1 h and washed, and nuclei were stained with DAPI as described. NF-κB/p65, NRF2, NQO1 and HO-1 fluorescence intensities in the nucleus were quantified using Image J 1.54g software as previously described [8].

### 4.4. Reverse Transcription−Real-Time Polymerase Chain Reaction

Total RNA was isolated from cells using a mi-Total RNA Isolation Kit (Metabion, Martinsried, Germany). Reverse transcription was performed with the use of random hexamer primers and MMLV (Moloney Murine Leukemia Virus) according to the manufacturer’s instructions (Fermentas, Vilnius, Lithuania). Prepared cDNAs were amplified by using Maxima SYBR Green/ROX qPCR Master Mix (Fermentas, Vilnius, Lithuania) according to the recommendations of the manufacturer in QuantStudio 3 (Applied Biosystems, Foster City, CA, USA). The thermocycler conditions comprised an initial step at 50 °C for 5 min, followed by a step at 95 °C for 10 min, and a subsequent two-step PCR program at 95 °C for 15 s and 60 °C for 60 s for 40 cycles. The PCR primers (Metabion, Martinsried, Germany) were as follows: *β-actin*: 5′-CCA GCG CAG CGA TAT CG-3′; 5′-GCT TCT TTG CAG CTC CTT CGT-3′; *Arg1*: 5′-CCT GCT GTC CTG TGA TAC CC-3′; 5′-CGG CTG TGC ATC ATA CAA CG-3′; *Gclc*: 5′-CAT CTA CCA CGC AGT CAA GG-3′; 5′-TCA TGA TCG AAG GAC ACC AA-3′; *Gclm*: 5′-ATG CTC CGT CCT TGG AGT T-3′; 5′-GCT GCT CCA ACT GTG TCT TG-3′; *Hmox1*: 5′-TAA GCT GGT GAT GGC TTC CT-3′; 5′-TCT GCT TGT TGC GCT CTA TC-3′; *Ido*: 5′-TGG GCT TTG CTC TAC CAC AT-3′; 5′-GGC AGC ACC TTT CGA ACA TC-3′; *Il10*: 5′- TGT GAA AAT AAG AGC AAG GCA GTG-3′; 5′-CAT TCA TGG CCT TGT AGA CAC C-3′; *Il23p19*: 5′–CAT GGG GCT ATC AGG GAG TA-3′; 5′- AAT AAT GTG CCC CGT ATC CA-3′; *Il27p28* 5′-TTC CCA ATG TTT CCC TGA CTT T-3′; 5′-AAG TGT GGT AGC GAG GAA GCA-3′; *Il6*: 5′-TAG TCC TTC CTA CCC CAA TTT CC-3′; 5′-TTG GTC CTT AGC CAC TCC TTC-3′; *Inos*: 5′-CTG CAG CAC TTG GAT CAG GA-3′; 5′-GCC AGA AAC TTG GGA AGG GA-3′; *Nqo1*: 5′-GAT CCT GGA AGG ATG GAA GA-3′; 5′-TCT GGT TGT CAG CTG GAA TG-3′; *Tnf*: 5′-CCA CGT AGC AAA CCA C-3′; and 5′-TGG GTG AGG AGC ACG TAG T-3′. PCR product accumulation was detected in real time, and for the analysis of the results, QuantStudio^TM^ Design&Analysis Software v1.4.3 (Applied Biosystems, Foster City, CA, USA) was used. The relative RNA expression is presented as 2^−dCt^, where dCt is the difference between Ct values of a gene of interest and the endogenous control (β-actin).

### 4.5. Statistics

The significance of the differences between the groups was determined via a *t*-test using GraphPad Prism version 9.00 for Windows (GraphPad Software, La Jolla, CA, USA).

## 5. Conclusions

The presented data imply that NRF2 signaling has a major role in the EP-imposed tolerogenicity of tEP-DCs. Also, our data suggest that EP should be added to the list of NRF2 activators. Our findings contribute to the understanding of the potent anti-inflammatory effect exerted in vivo in various animal models of chronic inflammatory disorders. To further elucidate the actions of EP in such in vivo settings, it will be important to investigate if NRF2 also plays a central role in the immunomodulatory influence of EP on T cells.

## Data Availability

Data are contained within the article. Additional information is available on request from the corresponding author.
